# Common features of neurodegenerative disease: exploring the brain-eye connection and beyond (Part 1): the 2021 pre-symposium of the 15th international conference on Alzheimer’s and Parkinson’s diseases

**DOI:** 10.1186/s13024-022-00570-8

**Published:** 2022-10-31

**Authors:** Sharyn L. Rossi, Preeti Subramanian, Guojun Bu, Adriana Di Polo, Todd E. Golde, Diane E. Bovenkamp

**Affiliations:** 1 BrightFocus Foundation, 22512 Gateway Center Dr, 20871 Clarksburg, MD USA; 2 Department of Neuroscience, Mayo Clinic, Jacksonville, FL USA; 3 Departments of Neuroscience and Ophthalmology, Centre de recherche du Centre Hospitalier de l’Université de Montréal (CRCHUM), University of Montreal, Montreal, QC Canada; 4 Departments of Pharmacology & Chemical Biology, and Neurology, Center for Neurodegenerative Disease, Emory University, School of Medicine, Atlanta, GA USA

**Keywords:** Alzheimer’s disease (AD) and related dementias, glaucoma, age-related macular degeneration (AMD), neurodegeneration, cellular senescence, senolytics, retinal ganglion cells (RGC), astrocytes, microglia, inflammation, longevity, geroscience, comorbid, biomarker, resilience, resistance, cognitive reserve, brain reserve, compensation

BrightFocus Foundation sponsored the third iteration of the ***“Common Features of Neurodegenerative Disease: Exploring the Brain-Eye Connection and Beyond”*** pre-conference symposium which has preceded the bi-annual **International Conference on Alzheimer’s and Parkinson’s Diseases (AD/PD)** for the past six years. *Common Features* highlights areas of disease pathology that share similar mechanisms of neurodegeneration across multiple diseases. This meeting report (Part 1 of 2) summarizes two of the hot topics discussed in the AD/PD 2021 *Common Features*, including cellular senescence and resilience/resistance (visit the meeting website for a complete agenda https://adpd2021.kenes.com/common-features-of-neurodegenerative-disease-exploring-the-brain-eye-connection-and-beyond). As funders of brain and eye diseases, BrightFocus supports research in each of these critical areas of disease pathology and anticipates that scientific discussions on these common elements will catalyze cross-disease collaborations and breakthroughs (information on how to apply for funding can be found at science.brightfocus.org/apply-grant). Since the topics and speakers change at every meeting, we look forward to your participation in the fourth installment of *Common Features* taking place at the AD/PD 2023 conference on March 28, 2023.

Alzheimer’s disease (AD) and related dementias, age-related macular degeneration (AMD), and glaucoma all are neurodegenerative diseases, with neuronal damages and losses in the eye or brain leading to functional impairment. The result can be side or central vision loss in glaucoma or AMD, or the onset of memory loss and dementia characteristic of AD.

BrightFocus Foundation is currently funding $75 million in 287 active science projects around the globe through its three programs: Alzheimer’s Disease Research, Macular Degeneration Research, and National Glaucoma Research. BrightFocus is leading the way to explore the common neurodegenerative features to bridge what was once thought of as disparate fields of science, leveraging the learnings of one disease to better inform and help accelerate research progress in another.

The 2021 program examined burning issues that unite diseases of mind and sight, and the lingering barriers to progress towards cures. Age is the single biggest risk factor that unites all three diseases of AD, AMD, and glaucoma. Accumulation of wear-and-tear on the nerves and supporting cells of the eyes and brain during aging may lead to common biological and biochemical features shared by these diseases. Lessons learned from tackling one disease could accelerate a solution to combat another. As such, this pre-symposium is a catalyst for future collaborations and scientific advances.

## Session I, “what is cellular senescence and what is its impact on neurodegenerative diseases of the brain and eye,” was chaired by Dr. Todd E. Golde

Cellular senescence takes place when cells transition into a permanent, irreversible state of cell cycle arrest that can reduce the function and regenerative potential of terminally-differentiated tissues while increasing a senescent-associated pro-inflammatory phenotype (SASP). Cellular senescence may be particularly important for diseases where age is the number one risk factor. Current research is focused on identifying specific genetic and proteomic signatures of senescent cells and developing novel approaches to remove senescent cells, decreasing their pro-inflammatory phenotype, or reverting them back to a proliferating state.

Darren Baker, PhD, Mayo Clinic, Rochester, focuses his research on novel treatment models to tackle senescence in AD. Senescence results in stem cell exhaustion, degradation of extracellular matrix molecules, and SASPs, where cells release a plethora of inflammatory molecules like cytokines, chemokines, proteinases, and growth factors. Senescent cells that express the cell cycle inhibitory protein, p16, can be targeted in animal models using gene therapy and senolytic drugs to activate the caspase pathway and induce apoptosis. Dr. Baker’s work has demonstrated that the primary senescent cells accumulating in the brain of PS19 tauopathy mice are astrocytes and microglia. These senescent cells promote insoluble tau aggregates and drive neurodegeneration. Clearance of senescent astrocytes and microglia using senolytic drugs attenuates tau phosphorylation and restores recognition memory [1].

Senescence can occur as a response of normal cells to persistent damage that becomes resistant to apoptosis. The cells then undergo various cellular and molecular changes, including SASPs that affect the cellular environment. Dorota Skowronska-Krawczyk, PhD, University of California, Irvine, observed senescent cells in the retinal ganglion cells (RGCs) of glaucomatous eyes from human donor samples [2] and further demonstrated the presence of more senescent cells in the eye of mouse models with acute increase in intraocular pressure (IOP). Interestingly, a retrospective safety evaluation performed on cohorts with ophthalmic data on senolytic drug exposures revealed no changes in IOP levels and no decrease in visual acuity. The absence of any adverse effects or ocular toxicity is reassuring of the safety of senolytic drugs for early stages of glaucoma.

The role of senescence in vascular diseases of the eye is being investigated by Przemyslaw (Mike) Sapieha, PhD, from the University of Montreal. Dr. Sapieha’s work on the oxygen-induced retinopathy model identified clusters of immune cells using single-cell RNA sequencing of the retina [3]. They detected more genes associated with neutrophils and senescent-associated genes at the peak of neovascularization. They showed that senescence factors secreted by the vasculature attract neutrophils and trigger the production of neutrophil extrusion traps (NETs). NETs play a role in removing senescent vasculature and restoring normalization. Furthermore, small-molecule inhibitors of the senescence pathway promoted regression and remodeling of vessels that may be beneficial to restoring normalization [4]. Thus, targeting senescent vasculature can be a promising treatment for retinopathies like proliferative diabetic retinopathy or neovascular AMD.

To provide a resource for geroscience researchers, Joao Pedro de Magalhaes, PhD, University of Liverpool, has developed an open database that identifies genetic signatures of cellular senescence in aging and longevity across different tissues and model organisms (Human Aging Genomics Resources https://genomics.senescence.info/about.html). This includes genes commonly altered during aging as well as a longevity map that serves as a repository for human genetic variants associated with longevity. Interestingly, using whole transcriptome sequencing, Dr. Magalhaes and colleagues identified many differentially expressed dark matter transcripts (transcripts that do not map to known exons) during aging [5]. This work demonstrates that dynamic changes in RNA are associated with aging that could not be identified using conventional microarrays.

## Where can we make an impact on cellular senescence and neurodegenerative disease?

An important question facing the field is how to identify senescent cells and distinguish them from post-mitotic cells. In the quest for a senescent cell biomarker, it may be possible to leverage the distinct metabolic profile that arises in senescent cells to not only identify senescent cells, but to better understand the intracellular mechanisms that underlie senescence. However, it is unclear whether the distinctive phenotypes of senescent cells in model systems recapitulate what occurs in human disease states. Having methods to identify and separate inflammatory signals from quiescent, reactive, and senescent cells will help to characterize cells across tissues and over time. To define a senescent cell, developing reliable biomarkers is critical, and efforts are underway through the NIH Cellular Senescence Network (SenNet: https://sennetconsortium.org/) program to generate a senescent cell atlas that will map the characteristics of different senescent cell types across different health states.

Pharmacologically, there are questions surrounding targeting senescent cells in acute or chronic states as acute senescence is often beneficial and induced due to stressors for a specific purpose, while post-mitotic cells like neurons are also in a state of senescence. While the use of senolytics in humans has only just begun, the consensus is that a transient clearance of senescent cells coupled with lifestyle interventions, thereafter, might be sufficient to ameliorate certain neurodegenerative diseases such as diabetic retinopathy. How this translates to lifelong progressive diseases such as AD is yet to be determined. Dr. Golde closed the discussion by asking how senescence plays a role in proteinopathies like AD. Does protein aggregation induce senescent phenotypes, is there a secondary stress associated mechanism that leads to senescence, or does senescence itself contribute to proteinopathy?

## Session II, “how does resilience, from genetics to environmental, at the cellular, systems, or population levels, play a role in reducing risk for neurodegenerative disease”, was chaired by Dr. Guojun Bu

The ability to escape disease despite the occurrence of risk factors or disease pathology is considered resilience, while resistance is the ability to avoid AD pathology entirely. Thomas Montine, MD, PhD, Stanford University, identified three main steps in the progression of neurodegenerative disease to clarify the temporal appearance of resistance versus resilience. A primary neuronal injury and response leads to secondary neuronal damage which manifests as cognitive impairment. The avoidance of secondary neuronal damage is resistance to disease while the lack of conversion to step three, cognitive impairment, confers resilience to disease.

To understand how different neuropathological features manifest cognitively and to determine the prevalence of resilience and resistance across the neuropathological spectrum, Dr. Montine utilizes a population-based study of longitudinal aging and a community cohort of people aged 90+. They found that the cognitive performance of those vulnerable to AD was not significantly different than those that are resistant but, those that are vulnerable to co-morbidities did significantly differ, indicating that the presence of one additional co-morbidity accounts for the severe cognitive impairments observed in the oldest old [6]. In terms of resilience, this shows that people with AD are more resilient to cognitive decline than those with multi-etiological dementias, reinforcing a “two hit” neuropathological prerequisite to severe cognitive impairment.

The most potent genetic risk factor for developing AD is the lipid transport gene *APOE*, where possessing one copy of the *APOE4* allele increases risk 3-4-fold. The *APOE2* allele is known to be protective against AD while the *APOE3* allele is neutral. Dr. Guojun Bu discussed evidence from a large study cohort that *APOE4* accelerates, and *APOE2* protects against, normal age-related memory decline as well as age of onset of AD. Even in healthy cohorts, increased levels of APOE2 confer resistance and increase lifespan, in part by promoting cholesterol efflux from the brain parenchyma into the CSF. Therefore, increased cholesterol efflux and metabolism by APOE2 promotes longevity and healthspan through effects on preserving activity [7].

Other mutations have also been identified that confer resistance to AD - particularly the APOE3-Christchurch mutation (R136S) and the APOE-Jacksonville (Jac) mutation (V236E), discovered by Dr. Guojun Bu [8, 9].

How do we know if someone will be resilient to AD or other related dementias? Dr. Rik Ossenkoppele, Amsterdam UMC, Netherlands, utilizes a statistical residual model of resilience to obtain individualized resilience scores that can be used as a predictive tool for cognitive trajectories and/or to correlate proxies of resilience (education, socioeconomic status, genomics) and tau PET imaging signatures. Cognitive reserve is the adaptability of cognitive processes (efficiency, capability, flexibility) while brain reserve is a neurobiological correlate that reflects the integrity of neural circuits. Cognitive and brain resilience may be associated with different mechanisms as a recent study that looked at tau pathology in clinically impaired individuals on the AD continuum, demonstrated that female sex and early age conferred greater brain resilience while higher education and global cortical thickness correlated with greater cognitive resilience [10].

In the eye, selective RGC types have been shown to have resilience to damage or injury. Steven Barnes, PhD, Doheny Eye Institute, discussed the functional and morphological diversity of alpha RGC subtypes and their ability to modulate electrophysiological responses in the presence of reactive oxygen species (ROS). Dr. Barnes showed that during increased endogenous ROS, the ON-sustained alpha RGCs spike less and the transient RGCs had the opposite response showing increased spiking. The OFF-sustained spike less during increased ROS, and OFF-transient also show a small effect. Thus, sustained RGCs are set to handle long phases of slow spiking. Furthermore, ROS modulates the sodium (Na) channel gating to have a lower threshold in transient cells, permitting more spikes, but further increase in ROS production causes cessation of spiking due to Na channel inactivation. Different alpha RGCs have differentially tuned Na current activation properties that contribute to varied spiking responses in response to ROS levels.

## How to translate knowledge on resistance, resilience, and reserve of neurodegenerative disease to therapy?

While mechanisms of resistance, resilience, and reserve have been studied for some decades, the lack of translation to a clinical setting has disadvantaged the field by neglecting to collect and provide valuable information for both basic and clinical studies. Resilience is driven by many factors including genetics, lifestyle, and environment, and it is important to understand risk factors and mechanisms of protection that can teach us how to treat disease. Recently, the Collaboratory on Research Definitions for Reserve and Resilience in Cognitive Aging and Dementia developed consensus definitions and research guidelines that should be used as standardized nomenclature for research on reserve and resilience (https://reserveandresilience.com/framework/). The ability to systematically identify people as resistant or resilient to disease across cohorts will allow for more segregated GWAS that can better identify genes critical for resistance and resilience. This will lead to better clinical trial cohort grouping, statistical analysis, and treatment outcomes, increased probability for the detection of sex differences in resistance and resilience and optimizing treatment regimens for a precision medicine approach.

**To Be Continued in Part 2 (Vascular Components and Conclusions)**.

## Data Availability

Not applicable.

## Abbreviations

Aβ: amyloid-beta/beta-amyloid
AD: Alzheimer’s disease
ALS: amyotrophic lateral sclerosis
AMD: age-related macular degeneration
CSF: cerebrospinal fluid
DR: diabetic retinopathy
FTD: frontotemporal dementia
GWAS: Genome-wide Association Studies
HAGR: Human Aging Genomics Resources https://genomics.senescence.info/about.html
IOP: intraocular pressure
p16: known as p16^INK4a^, cyclin-dependent kinase inhibitor 2 A, CDKN2A, multiple tumor suppressor 1 and numerous other synonyms
PD: Parkinson’s disease
PET: positron emission tomography
RGC: retinal ganglion cells
ROS: reactive oxygen species
SASP: senescence associated secretory phenotype
SenNet: NIH Cellular Senescence Network https://sennetconsortium.org/
TDP-43: TAR DNA binding protein 43
